# Pediatric Hospital Medicine Resident Elective: A Novel Resident Curriculum for an Evolving Field

**DOI:** 10.7759/cureus.23451

**Published:** 2022-03-24

**Authors:** Katherine O Salada, Victoria A Rodriguez, Zarina Norton, Kathryn L Jackson, Robyn A Bockrath

**Affiliations:** 1 Pediatric Hospital Based Medicine, Ann & Robert H Lurie Children’s Hospital of Chicago, Chicago, USA; 2 Pediatrics, Northwestern University Feinberg School of Medicine, Chicago, USA; 3 Medical Social Sciences, Northwestern University Feinberg School of Medicine, Chicago, USA

**Keywords:** curriculum evaluation, kern's six-step framework, curriculum development, pediatric resident elective, pediatric hospital medicine

## Abstract

Background

Although 8-10% of pediatric residents pursue a career in Pediatric Hospital Medicine (PHM), many report an incomplete understanding of PHM careers and would benefit from a PHM elective.

Methodology

We followed Kern’s six-step curriculum development framework. A general needs assessment via literature review revealed a lack of published PHM elective curricula. A targeted needs assessment was conducted by surveying national PHM fellowship program directors, national PHM fellows, local junior PHM attendings, and local pediatric residents. Content analysis from these surveys was used to develop a PHM resident elective curriculum. The curriculum was implemented and evaluated through an experience log and written reflections.

Results

Needs assessment surveys were completed by fellowship directors (22/61, 36%), fellows (36/103, 35%), attendings (10/26, 38%), and residents (15/98, 15%). Common themes included the importance of academic experiences, mentorship, non-teaching and non-inpatient clinical experiences, community hospital experience, and the desire to address knowledge gaps. Significant variability in survey responses suggested the importance of an individualized curriculum. Goals, objectives, and aligned educational strategies were developed to provide a breadth of clinical experiences, mentorship, and PHM-focused academic activities, with an emphasis on individualization. Implementation of the curriculum began in July 2021 and four residents enrolled in 2021-2022. The curricular evaluation demonstrated the achievement of objectives and improved resident awareness of PHM opportunities, clinical skill development, ancillary shadowing, and academic opportunities.

Conclusions

A PHM resident elective was developed using Kern’s six-step approach with input from national fellows and fellowship program directors to address educational gaps and increase exposure to PHM careers. The next steps include the evaluation of the impact of the PHM elective on career choice and preparedness of residents.

## Introduction

Over the last 20 years, the field of Pediatric Hospital Medicine (PHM) has been steadily growing and evolving. Historically, outpatient pediatricians followed their patients both in and out of the hospital [1]; however, a 2012 American Academy of Pediatrics survey showed that outpatient pediatricians are the inpatient attending for less than one-third of admitted patients from their practice [2]. Hospitalist services continue to expand and now dominate in large children’s hospitals, with 98% of hospitals associated with academic pediatric departments reporting dedicated PHM hospitalist services [2]. The American Board of Medical Specialties approved PHM as a pediatric subspecialty in 2016, and PHM was approved as a two-year Accreditation Council for Graduate Medical Education (ACGME) pediatric fellowship in 2020. Today, PHM is one of the largest areas of practice for graduating pediatric residents, with 8-10% of graduating residents pursuing a hospital medicine career [3-5]. With the recent creation of a PHM ACGME fellowship and board exam, PHM educational environments must continue to evolve to meet the specialty’s changing professional identity.

The ACGME requires that pediatric residents spend a minimum of 10 months on inpatient care, with at least five months dedicated specifically to inpatient general medicine and subspeciality rotations to learn the practice of hospital medicine [6]. However, a hospital medicine attending career is more diverse than what residents typically experience. PHM is an academic field where providers can mold their career and balance clinical time, medical education, research, administrative leadership, or a combination of these areas. Hospitalists also work in varied clinical settings, many of which are not affiliated with pediatric residency rotations (e.g., attending only inpatient services or sedation services). Despite spending substantial time on inpatient medicine rotations, without exposure to the breadth of the PHM field, residents risk graduating with an incomplete understanding of a pediatric hospitalists’ career.

A PHM residency elective offers the opportunity to educate residents about a hospitalist career and expose them to hospital medicine fellows and attendings. At our 300-bed academic free-standing children’s hospital, a PHM resident elective existed previously; however, it was under-utilized (between zero and two residents enrolled yearly) and not inclusive of new PHM fellowship opportunities. National trends in PHM elective curricula within residency programs are unknown, the current literature lacks any recently published PHM elective curriculum, and the curricula that do exist likely vary widely given the recent developments in the field of hospital medicine.

There is also minimal data about new hospitalists or fellow perspectives on PHM resident electives, though studies have examined areas of perceived deficits and strengths in training and career preparedness. One study found that while most new hospitalists feel that residency provided adequate clinical skills and communication training, quality improvement (QI) methods and administration duties are areas of inadequate training [7]. Early career non-fellowship trained hospitalists have reported feeling less competent in sedation, business practice, phlebotomy, and plan-do-study-act and root cause analysis work [8]. Additionally, most hospitalists desired additional training in medical education to enhance their skills and knowledge in teaching medical students and residents [9]. We suspect these areas of perceived weaknesses translate to potential needs in a PHM elective; however, this data has not yet been directly studied concerning a PHM resident elective.

Published literature on PHM resident elective curriculum development is scarce. Indiana University created a PHM elective and shared their curriculum material on MedEdPortal in 2009 [10]; however, the data used to create or evaluate the curriculum was not included in their abstract and the curriculum pre-dates PHM as an accredited sub-specialty. No literature on PHM resident elective curriculums is available from other institutions, and the field of PHM has evolved considerably since the original curriculum publication. Given the expanding PHM field, we sought to create a PHM elective curriculum that increases elective participation, addresses the needs of trainees by filling individualized knowledge gaps, and improves resident preparedness for roles as future PHM fellows and attendings.

This article was previously presented in part as a meeting abstract at the Central Group on Educational Affairs Association of American Medical Colleges virtual regional meeting (March 2022).

## Materials and methods

We followed Kern’s six-step approach to curriculum development: (1) General needs assessment; (2) targeted needs assessment; (3) goals and objectives; (4) educational strategies; (5) implementation; and (6) evaluation and feedback. A general needs assessment was performed through a literature review, which revealed a lack of published PHM elective curriculum models. This study was approved with a waiver of consent by our local institutional review board (IRB #2021-4442).

Targeted needs assessment

A targeted needs assessment was conducted by surveying categorical pediatric residents at a single academic institution, pediatric hospitalists in their first or second year of post-residency practice (junior hospitalists) at the same institution, national PHM fellows, and national PHM fellowship program directors in May 2021. This group of respondents was chosen to investigate and contrast the opinions among individuals with different levels of training in the field of PHM. All local categorical pediatric residents (PGY 1-3) were included to elicit opinions on what residents consider generally important in electives, as well as specifically in a PHM elective. Local junior attendings (one to two years post-residency) were surveyed to capture the opinions of those who are still transitioning into the role of attending. Only junior attendings who did not complete a PHM fellowship were included, allowing them to provide insight regarding gaps in their pediatric residency training that a PHM elective may have filled to better prepare them for their role as an attending. As part of the junior hospitalist survey, participants were asked if they would like to be involved with the PHM elective (through mentorship, teaching, feedback, or shadowing) as a means of recruitment for faculty participation in the elective. Although more senior attendings (greater than two years post-residency) were not surveyed due to concern regarding their recall accuracy in identifying early-career knowledge and skill gaps; they were later recruited for mentorship participation in the elective (see “curriculum development and implementation” below). National PHM fellows were surveyed to elicit opinions on what elective experiences would increase preparedness for PHM fellowship. Finally, national PHM fellowship program directors were surveyed to elicit insight about what leaders in the field of PHM believe should be included in PHM electives to prepare trainees for PHM fellowship and data on what PHM elective curriculums currently exist at institutions nationally.

Local pediatric residents, local junior hospitalists, and national PHM program directors were contacted by email for recruitment, with a follow-up reminder email sent two weeks later. National PHM fellows were contacted via the PHM fellows group chat using the application WhatsApp. Email scripts and a scripted WhatsApp message contained an electronic link to one of four surveys. Participation was voluntary and participants were free to opt-out of the study at any time. All survey answers were completely anonymous.

Statistical analysis

All four surveys were completed using the software Qualtrics. Descriptive statistics examined the frequencies and percentages for categorical variables, including demographic information, existing elective knowledge/details, and ideal elective logistics. Average results for impact on elective choice, preferences in elective experiences, and preferences for learning modality were calculated. For specific elective experience and learning modality preferences, respondents indicated their top four (ranked) priorities; for analysis, the first ranked (top) priority was given a score of four, and the fourth (lowest) priority was given a score of one; those not indicated as a priority were given a score of zero. Differences between groups were assessed using analysis of variance for normally distributed outcomes and Kruskal-Wallis tests for non-normal outcomes. A p-value of <0.05 was assigned to denote significance. Qualitative data from free-response questions were analyzed for major themes by two investigators using content analysis. All analyses were completed using SAS version 9.4 (SAS Institute Inc., Cary, NC).

Curriculum development and implementation

**Figure 1:**
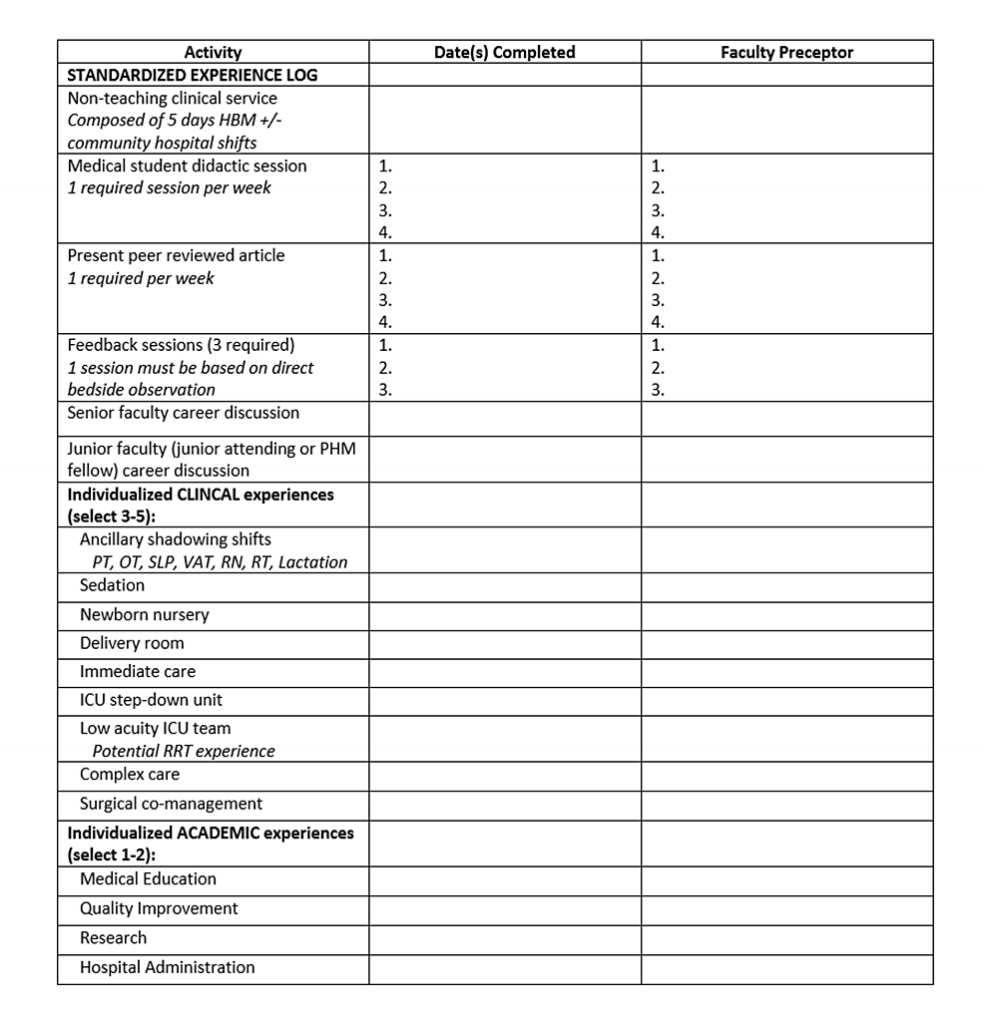
Experience log for residents to document clinical and academic experience. ICU = Intensive Care Unit; PT = physical therapy; OT = occupational therapy; SLP = speech and language pathology; VAT = Vascular Access Team; RN = Registered Nurse; RT = respiratory therapy; RRT = Rapid Response Team

**Figure 2:**
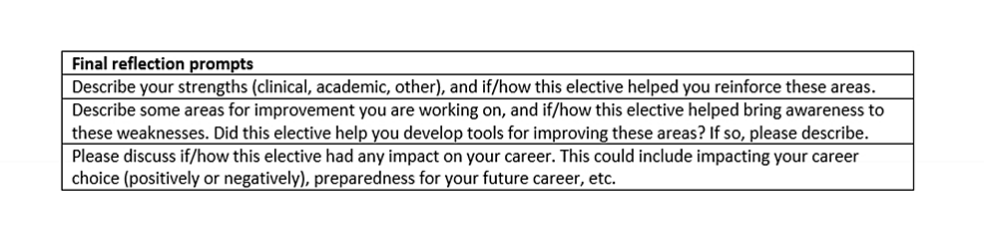
Prompts used for completing a written reflection after completion of each elective.

Goals, objectives, and aligned educational strategies (Tables 1, 2) were developed based on the themes identified in the general and targeted needs assessment. A PHM resident elective was piloted for categorical second- and third-year pediatric residents at a single academic center in July 2021. To enroll, residents freely expressed their desire to participate in the PHM elective to elective leadership at the start of the academic year. Interested residents then communicated with elective leadership via phone regarding their individualized interests to cater the experience to each learner. Experiences were scheduled and arranged according to each learner’s desired duration of elective participation (two to four weeks). Local junior hospitalists who indicated an interest in being involved during our targeted needs assessment, senior hospitalists, and PHM fellows were recruited to engage in mentorship meetings and feedback sessions with resident participants. Residents were required to document completion of their elective experiences in a log, demonstrating they met requirements for standardized elective content, as well as the minimum number of individualized curriculum selections (three to five clinical experiences and one to two academic experiences; Figure 1). Residents additionally completed a written reflection (Figure 2) and debriefing with the elective leader following completion of the elective to provide any additional comments or feedback regarding their experience.

**Table 1:** Curriculum goals. PHM = Pediatric Hospital Medicine

Curriculum goals
Fill clinical/educational gaps in the area of PHM within the pediatric residency curriculum
Provide an opportunity for increased preparedness for the specialty of PHM
Expose residents to academic aspects of a pediatric hospitalist’s career
Expand interest in PHM
Standardize a structure for the PHM residency elective, with the opportunity for individualization
Provide faculty mentorship for residents interested in careers in PHM

**Table 2:** Objectives with aligned educational strategies and evaluation methods. ICU = Intensive Care Unit; PHM = Pediatric Hospital Medicine

Objective (On their PHM elective, pediatric residents will…)	Educational method	Evaluation method
Complete one week as the front-line provider on a non-teaching clinical service (attending only hospitalist service and/or community hospital)	Demonstration; clinical experiences	Experience log
Directly care for inpatient pediatric patients and receive ≥3 verbal feedback sessions with pediatric hospitalists and/or PHM fellows on clinical skills and reasoning	Clinical experiences; reflection on experience	Experience log; faculty feedback
Meet with ≥2 pediatric hospitalists (minimum of one senior AND one junior faculty/PHM fellow) for career advising	Role models; reflection on experience	Experience log; reflective discussion with faculty
Lead one educational session for medical students per week on a general pediatric inpatient medicine topic	Peer teaching; discussion, small group	Experience log
Demonstrate use of evidence-based medicine by presenting one peer-reviewed journal article per week at the PHM division meeting	Readings; discussion, large group	Experience log
Participate in an individualized CLINICAL curriculum by choosing 3-5 of the following experiences: ancillary shadowing shifts, sedation medicine, newborn nursery, delivery room, immediate care, low acuity hospitalist ICU team, complex care, surgical co-management	Readings; demonstration; role models; clinical experiences	Experience log
Participate in an individualized ACADEMIC curriculum by choosing 1-2 of the following experiences: medical education, quality improvement, research, hospital administration	Lectures; clinical experiences	Experience log
Complete an experience log and provide written reflection with key learning points from the elective, with a focus on strengths, areas for improvement, and impact on career development	Reflection on experience	Written reflection form

## Results

Descriptive data from the survey

Surveys were completed by pediatric residents (15/98, 15%), junior hospitalists (10/26, 38%), PHM fellows (36/103, 35%), and PHM fellowship program directors (22/61, 36%). Most program directors who completed the survey (21/22, 95%) reported the existence of a PHM elective at their institution, with the majority reporting either zero to three (15/22, 68%) or four to six (3/22, 14%) yearly participants (Table 3). If a PHM elective was offered at their institution, 13/16 (81%) fellows and 4/7 (57%) junior hospitalists reported that they had completed the elective (Table 4). Data from program director surveys about existing PHM electives nationally reported 27% individualized curriculums, 50% combined standardized + individualized curriculums, and 22% standardized curriculums (Table 3).

**Table 3:** Existing PHM residency electives nationally. NA = not applicable; PHM = Pediatric Hospital Medicine

	National Program Directors (n = 22)
Total number of residents in the program	
0–49	9 (40.9%)
50–99	9 (40.9%)
100–150	4 (18.2%)
PHM elective currently offered	
Yes	21 (95.5%)
No	0 (0.0%)
In the process of creating	1 (4.5%)
Number of residents enrolled in elective per year	
0–3	15 (68.2%)
4–6	3 (13.6%)
7–9	1 (4.5%)
10–12	2 (9.1%)
NA	1 (4.5%)
Existing curriculum design	
Individualized	6 (27.3%)
Standardized	5 (22.7%)
Combined (individualized + standard)	11 (50.0%)
PHM elective makes fellowship applicants more favorable	
Yes	8 (36.3%)
No	8 (36.3%)
Unsure	6 (27.3%)

**Table 4:** PHM residency elective experience details. Resident respondents were asked what they want in a PHM elective, whereas fellows and hospitalists were asked what their experience was during a PHM elective if they took one previously. NA = not applicable; PHM = Pediatric Hospital Medicine

	Local residents (n = 15)	Local hospitalists (n = 10)	National fellows (n = 36)
Offer PHM Elective			
Yes	6 (40.0%)	7 (70.0%)	16 (44.4%)
No	9 (60.0%)	3 (30.0%)	19 (52.8%)
Unknown	0 (0.0%)	0 (0.0%)	1 (2.8%)
PHM elective completed			
Yes, took elective		4 (40.0%)	13 (36.1%)
Yes, intend to take	2 (13.3%)		
Did not take elective		3 (30.0%)	6 (16.7%)
Do not plan to take	6 (40.0%)		
Undecided	7 (46.7%)		
NA		3 (30.0%)	17 (47.2%)
PHM elective helpful			
Very helpful	3 (20.0%)	2 (20.0%)	7 (19.4%)
Somewhat helpful	9 (60.0%)	2 (20.0%)	6 (16.7%)
Not at all helpful	3 (20.0%)	0 (0.0%)	0 (0.0%)
NA	0 (0.0%)	6 (60.0%)	23 (63.9%)
Hours per week			
<20	1 (6.7%)	0 (0.0%)	0 (0.0%)
20–30	0 (0.0%)	0 (0.0%)	1 (2.8%)
30–40	11 (73.3%)	4 (40.0%)	6 (16.7%)
40–50	2 (13.3%)	0 (0.0%	4 (11.1%)
50–60	1 (6.7%)	0 (0.0%)	1 (2.8%)
>60	0 (0.0%)	0 (0.0%	1 (2.8%)
NA	0 (0.0%)	6 (60.0%)	23 (63.9%)
Elective year desired			
Second year	3 (20.0%)		
Third year	12 (80.0%)		
Duration desired			
One week	3 (20.0%)		
Two weeks	11 (73.3%)		
Three weeks	0 (0.0%)		
Four weeks	1 (6.7%		

Despite the existence of a local PHM elective, 9/15 (60%) resident respondents did not know an elective was offered at their institution (Table 4). There was consensus among residents, fellows, and junior hospitalists that an average workweek of 30-40 hours was appropriate for the PHM elective (Table 4). The majority of residents felt they would benefit most from taking the elective during their third year (12/15, 80%), with a preferred duration of two weeks (11/15, 73%). A large proportion of resident respondents (40% yes, 33% unsure) were considering a career in PHM, with 13% planning to apply for a PHM fellowship (47% unsure).

Targeted needs assessment based on survey responses

Residents, hospitalists, and fellows all reported that addressing gaps in knowledge was the main motivation in selecting a PHM elective, followed by networking and maintaining quality of life during the elective (Table 5). There was a statistically significant difference between groups on their prioritization of inpatient general pediatric medicine clinical experiences. Program directors reported that the most important factor for preparing future PHM fellows was inpatient general medicine clinical experience, while residents, hospitalists, and fellows valued other clinical experiences more highly, such as sedation, delivery room resuscitation, and urgent care (p < 0.001; Table 6). All four surveyed groups valued academic experiences relevant to PHM (e.g., medical education, research, and QI), with fellows and program directors valuing these experiences more than residents and hospitalists (p = 0.002; Table 6).

**Table 5:** Factors influencing elective choice. Mean impact of survey response group rated on a seven-point scale (7 = most important, 1 = least important) with standard deviation.

	Residents (n = 15)	Hospitalists (n = 10)	Fellows (n = 36)	P-value for difference
Letter of recommendation	1.80 ± 0.7	1.44 ± 0.5	1.43 ± 0.7	0.132
Address gaps in education	2.87 ± 0.4	2.67 ± 0.5	2.51 ± 0.7	0.161
Networking	2.40 ± 0.5	2.33 ± 0.7	2.11 ± 0.8	0.440
Quality of life	2.60 ± 0.5	2.56 ± 0.5	2.20 ± 0.7	0.125

**Table 6:** Preferences for clinical and non-clinical experiences in a PHM elective. ^a^Mean priority of survey response group, all choices rated on a seven-point scale (7 = most important, 1 = least important). ^b^Includes sedation, newborn, urgent care, etc. ^c^Includes research, QI, medical education, administration, etc. ^d^Includes networking, letter of recommendation, etc. ^e^Mean preferences of the survey response group, top four choices selected, rated on a four-point scale (4 = highest priority, 1 = lowest priority; 0 = not indicated as priority). ^f^Includes PT, OT, RT, RN, lactation, VAT, etc. ICU = Intensive Care Unit; OT = occupational therapy; PHM: Pediatric Hospital Medicine; PT = physical therapy; QI = quality improvement; RN = Registered Nurse; RT = respiratory therapy; VAT = Vascular Access Team

Experience	Residents (n = 15)	Hospitalists (n = 10)	Fellows (n = 36)	Program directors (n = 22)	P-value for difference
General experiences^a^					
Inpatient general medicine	3.07	2.63	3.91	6.43	<0.001
Non-general medicine clinical^b^	6.07	6.38	5.28	4.14	<0.001
Procedural	5.07	2.88	2.69	2.86	<0.001
Academic^c^	4.40	4.75	5.31	6.10	0.002
Career development^ d^	4.13	4.25	4.78	4.67	0.338
PHM career fit	3.87	5.38	4.81	2.81	0.092
Specific experiences^e^					
Inpatient teaching service	0.20	0.70	0.97	2.14	0.007
Inpatient non-teaching service	1.87	1.70	2.11	1.86	0.872
ICU	0.20	0.00	0.19	0.77	0.038
Newborn	0.07	0.90	0.14	0.45	0.038
Delivery	0.27	1.60	0.19	0.09	<0.001
Community hospital	2.80	3.00	1.75	1.36	0.003
Immediate care	0.60	0.20	0.25	0.32	0.403
Medical education	0.73	0.60	1.31	1.00	0.193
Administrative	0.40	0.40	1.69	0.82	0.019
Sedation	1.13	0.00	0.42	0.09	0.002
Ancillary shadowing^f^	1.07	0.90	0.39	0.09	0.020

Residents, hospitalists, and fellows preferred additional non-teaching inpatient general medicine clinical experience (e.g., on an attending-only service), whereas program directors prioritized clinical experience on an inpatient teaching service to prepare trainees for PHM fellowship (Table 6). Community hospital experience was desired by all groups, with residents and hospitalists placing greater priority in this area (p = 0.003; Table 6). Results showed a statistically significant difference among groups in the perceived value of various specific clinical experiences, such as delivery room (p < 0.001) and sedation (p = 0.002; Table 6).

There was a significant difference among respondent groups in the value placed on hospitalist (p < 0.001) and PHM fellow (p = 0.023) mentorship, with fellows and program directors prioritizing these opportunities (Table 7). There was also a significant difference in the value placed on procedural experience (p < 0.001) and procedural simulation exposure (p < 0.001), with residents valuing these experiences more than other groups (Tables 6, 7).

**Table 7:** Learning modality preference. ^a^Mean preferences of survey response group, top four choices selected, rated on a four-point scale (4 = highest priority, 1 = lowest priority; 0 = not indicated as priority). PHM = Pediatric Hospital Medicine; QI = quality improvement

Learning modality	Residents (n = 15)^a^	Fellows (n = 36)^a^	Program directors (n = 22)^a^	P-value for difference
Hospitalist mentorship	1.40	3.33	2.68	<0.001
PHM fellow mentorship	1.07	2.42	2.32	0.023
Lectures	0.07	0.08	0.18	0.974
Procedural simulation	1.20	0.19	0.00	<0.001
Clinical simulation	0.40	0.44	0.32	0.680
Feedback on clinical skills	1.20	0.72	1.23	0.288
Feedback on teaching skills	0.60	1.17	1.14	0.105
Clinical hospitalist experience	1.73	0.36	0.77	0.061
QI experience	0.40	0.78	1.00	0.274

Design of PHM curriculum goals, objectives, and educational strategies

Curriculum goals were developed to focus on addressing major themes from the general and targeted needs assessments (Table 1). Objectives with aligned educational strategies (Table 2) were developed to create a standardized curriculum that included inpatient non-teaching general pediatric clinical experience (community hospital vs. attending-only service), feedback, and mentorship. A significant portion of the curriculum was individualized, with customizable clinical and academic opportunities, allowing the elective to be tailored to the specific learner. These opportunities included choosing three to five clinical experiences (options: ancillary shadowing, sedation, newborn nursery, delivery room, immediate care, intensive care unit (ICU) step-down unit, low-acuity hospitalist ICU team with potential rapid response team (RRT) participation, complex care, surgical co-management) and one to two academic experiences (options: activities related to medical education, QI, research, hospital administration).

Implementation

Implementation of the PHM resident elective curriculum began as a pilot program in July, 2021. Four residents completed the elective for the academic year 2021-2022. Notably, no residents had enrolled in the previously offered PHM resident elective for the academic year 2020-2021.

Evaluation and feedback

Evaluation of the curriculum is ongoing via an experience log and written reflection form (Figures 1, 2). Four resident participants have completed the elective, experience log, and written reflection. All of these participants completed the required (standardized) experiences as tracked by their experience log. For the individualized experiences, resident clinical choices included: hospitalist ICU team (n = 4), complex care (n = 4), ancillary shadowing (n = 3), sedation (n = 2), newborn/delivery (n = 2), and immediate care (n = 1). Ancillary shadowing resident choices included working with the vascular access team (VAT; n = 2), occupational therapy (n = 1), and lactation (n = 1). For the individualized academic elective experience, three residents chose medical education and one resident chose QI.

The four PHM resident elective participants also completed a written reflection (Figure 1) to evaluate the impact of the new curriculum on personal strengths, areas for improvement, and career choice. Recurring themes from resident reflections included noting the benefits of exposure to the breadth of PHM career choices, clinical skill development, value of ancillary shadowing, exposure to care at alternative sites, and academic PHM experiences (Table 8). There was a paucity of comments regarding the value of mentorship and feedback exposure during the elective. Resident participants also met with elective leadership for a verbal debrief following the completion of the PHM elective. Themes that arose in debrief included desire for increased exposure to community medicine, the value of spending time with non-teaching clinical services, and the value of shadowing ancillary staff. In response to resident feedback, updates to the elective included additional ancillary shadowing experiences (respiratory therapy (RT) and lactation), as well as future plans to expand opportunities for community hospital exposure.

**Table 8:** Reflection themes from resident free response answers. PHM = Pediatric Hospital Medicine; QI = quality improvement; VAT = Vascular Access Team

Elective topic	Resident quote
Breadth of PHM career options	“[PHM] is an experience that each individual attending can make their own which is very appealing as a career path. It was also amazing to … hear about how they were able to integrate research, QI, or med ed into their careers”
Clinical skills development	“I watched attendings discuss amongst themselves and each other the differential/workup for their patients. I observed them thinking through their patient’s step by step and asking for help when they did not know where to go next. Observing and participating in this process reinforced these skills and highlighted the collaborative environment within this field”
Benefits of ancillary shadowing	“Seeing how [lactation and VAT] consults work will make me be able to place consults more appropriately and allocate resources more efficiently”
Differences in care at alternative sites	“Complex care [at another site] was particularly interesting because it taught me how to be resourceful and creative in situations where sometimes resources are limited (especially compared to [the main] hospital)”
Benefits of participating in academic PHM experiences	“Teaching the medical students while on the elective helped to reinforce my passion for teaching, but also gave me the opportunity to hone in on my skills … within academic medicine”
General positive feedback for the elective	“[…the rotation] made a very positive impact on my future career path”

## Discussion

An individualized PHM resident elective was developed using Kern’s six-step approach with input from national PHM fellows and program directors, as well as local residents and hospitalists. Although our needs assessment revealed the existence of PHM elective curricula at multiple institutions nationally, only one PHM elective from 2009 was published [10]. Our curriculum is the first to incorporate updated trends in PHM, novel data from local and national needs assessments, methods for implementation of the curriculum, and the use of an experience log for eliciting evaluation and feedback from participants. Based on a targeted survey-based needs assessment, the new PHM elective curriculum reflects input from stakeholders across the PHM educational continuum, from residents to fellowship program directors. Significant differences in preferences for elective experiences between survey response groups supported the creation of a curriculum that balances high-priority required content with robust customized opportunities to meet the needs of the individual resident. Initial data from curricular evaluation and feedback provided in completed experience logs revealed that the curriculum increased participation in the PHM elective, achieved learning objectives, addressed individualized educational gaps, and increased exposure to a career in PHM.

Limitations

This study was limited by a low response rate to the survey-based needs assessment across all categories. The response rate was the lowest among residents, likely reflecting participation by those most interested in pursuing a PHM elective and/or career. Similarly, pilot implementation and curriculum evaluation included a small sample size, which limits the generalizability of the results; however, the number of residents in the pilot is consistent with national trends in the typical number of yearly PHM elective participants, and the initial findings support a continuation of this work. While resident participants achieved the curricular objectives and reported a satisfactory experience, possible implications of the elective experience on educational outcomes (e.g., clinical skills evaluations, increased knowledge on PHM topics) and career choices (e.g., decision to enter the field of PHM) require future evaluation.

Next steps

The PHM resident elective will continue recruiting and enrolling residents and soliciting feedback on the curriculum. Using ongoing elective evaluation in the form of an experience log and written reflection, the curriculum will continue undergoing modifications to ensure it meets the educational needs of trainees. Further investigation is required to assess the longer-term impacts of a PHM resident elective on career choice and preparedness.

## Conclusions

While specific experiences may differ between institutions, this new PHM elective was designed with sound curricular methodology and input from national PHM fellows and leaders via targeted needs assessment, which increases its generalizability. The curricular framework can be piloted at other academic institutions, with the tailoring of experiences based on minor institutional variations. The investigators aim to support the implementation of this curriculum at other institutions, with the goal of further improving the experience based on ongoing feedback from a larger number of participants.
